# The impact of genital self-image on sexual function and sexual satisfaction in married women

**DOI:** 10.1590/1806-9282.20250935

**Published:** 2025-12-15

**Authors:** Yurdagül Günaydin, Esma Kir, Serpil Toker, Medine Kir Deprem

**Affiliations:** 1 Tarsus University, Faculty of Health Sciences, Department of Nursing – Mersin, Turkey.; 2 Yozgat Bozok University, Faculty of Health Sciences, Department of Midwifery – Yozgat, Turkey.; 3 Tokat Gaziosmanpaşa University, Faculty of Health Sciences, Department of Midwifery – Tokat, Turkey.; 4 Kayseri City Training and Research Hospital, Department of Family Medicine – Kayseri, Turkey.

**Keywords:** Body image, Sexual behavior, Sexual satisfaction, Women

## Abstract

**OBJECTIVE:**

This study was conducted to determine the effect of genital self-image on sexual function and satisfaction in married women.

**METHODS:**

The study included 353 married women in Central Anatolia. Data were collected using the Descriptive Information Form, the Female Genital Self-Image Scale, the Female Sexual Function Index-6, and the Golombok-Rust Inventory of Sexual Satisfaction.

**RESULTS:**

The participants’ mean total scores were 20.36±4.03 on the Female Genital Self-Image Scale, 19.46±4.98 on the Female Sexual Function Index-6, and 55.84±16.79 on the Golombok-Rust Inventory of Sexual Satisfaction. The regression analysis indicated that Female Sexual Function Index-6, age, marriage duration, and total Golombok-Rust Inventory of Sexual Satisfaction scores collectively predicted Female Genital Self-Image Scale scores, accounting for 13.2% of variance and suggesting a modest yet meaningful effect.

**CONCLUSION:**

Approaches that enhance genital self-image support women's sexual health, and it is essential for healthcare professionals to provide quality care by taking these factors into consideration.

## INTRODUCTION

Body image refers to how individuals perceive their physical appearance and the thoughts and feelings associated with that perception, shaped by cultural, social, psychological, and personal factors^1^. Within this framework, genital self-image (GSI) is a distinct dimension of body image, encompassing esthetic, functional, and emotional genital perceptions^2^. As a related construct, sexual satisfaction encompasses the cognitive, emotional, and relational fulfilment derived from sexual experiences. In women, GSI demonstrably shapes both sexual satisfaction and sexual function^2–4^. Positive GSI enhances body perception and lowers anxiety, whereas negative GSI is linked to sexual dysfunction and impaired orgasm. Sexual function includes desire, arousal, lubrication, orgasm, and pain: maladaptive genital attitudes can diminish desire and arousal^2–4^.

This framework robustly integrates Objectification Theory and Cognitive-Behavioral Models. According to the Objectification Theory, women are socialized to evaluate their bodies, including their genitalia, from the perspective of an external observer, fostering body surveillance, shame, and anxiety that negatively affect sexual well-being^5^. Cognitive-Behavioral Models emphasize that maladaptive cognitions, negative genital self-perceptions, and attentional biases reduce sexual desire, arousal, and satisfaction. Recent evidence indicates that negative GSI disrupts sexual pleasure via increased distraction and anxiety during sexual activity^4,6^. Thus, GSI functions as a cognitive-emotional schema shaping women's sexual health.

Low sexual satisfaction can contribute to sexual dysfunction over time, with GSI playing a determining role^7,8^. Healthy GSI correlates positively with sexual activity and orgasm frequency^2,7^, whereas negative GSI is associated with sexual avoidance, shame, anxiety, and dissatisfaction^4^. Negative genital body image adversely impacts women's emotions, thoughts, and behaviors, thereby diminishing sexual quality of life^2–8^. A proxy for this trend is the rising demand for genital cosmetic procedures^8^. Internalizing societal genital ideals may impair self-esteem and sexual function early. The aim of this study was to determine the impact of GSI on sexual function and sexual satisfaction in married women.

## METHODS

### Study design

The research is descriptive and cross-sectional.

### Study setting

The research was conducted between April and October 2024 in a province located in the Central Anatolia Region.

### Sample size and participants

The study population comprised women aged 18–60 years from a province in Central Anatolia, including married women living with their spouses, who actively used smartphones and social media, and who voluntarily consented to participate. Although this may exclude some rural or offline populations, the widespread smartphone use among literate women in Turkey minimizes potential bias. Nonconsenting participants and incomplete responses were excluded, and missing data were minimal, posing no threat to study validity. The sample size was calculated using G*Power 3.1.9.7 based on Cohen's^9^ medium effect size, 5% error margin, and 80% power^10^, with 351 married women completing the study. Out of 371 participants, 20 with incomplete responses were excluded, leaving 351 questionnaires for analysis with minimal risk of bias.

### Data collection tools

The data were collected using the Descriptive Information Form, the Female Genital Self-Image Scale (FGSIS), the Golombok-Rust Inventory of Sexual Satisfaction (GRISS), and the Female Sexual Function Index (FSFI-6).

### Descriptive Information Form

The researchers developed this form based on the literature^4,8^ and expert opinions.

### Female Genital Self-Image Scale

FGSIS is a seven-item, four-point Likert scale assessing women's GSI, validated in Turkish by Ellibes Kaya et al^11^. Scores range from 7 to 28, with higher scores indicating a more positive self-image. Cultural adaptation involved translation, review, pretesting, and finalization. Cronbach's alpha was 0.81 in the original study^11^ and 0.82 in our study.

### Female Sexual Function Index

FSFI-6, validated in Turkish by Sonbahar et al.^12^, is a six-item scale ranging from 2 to 30, with lower scores indicating dysfunction. The Turkish adaptation followed standard translation and validation procedures, ensuring cultural and linguistic validity. Cronbach's alpha was 0.86 in the Turkish study^12^ and 0.81 in the current study, confirming reliability.

### Golombok-Rust Inventory of Sexual Satisfaction

GRISS, a 28-item five-point Likert scale validated in Turkish by Tuğrul et al.^13^, ranges from 0 to 140, with higher scores indicating lower sexual satisfaction. The Turkish adaptation ensured conceptual and cultural-linguistic validity through translation, pretesting, back-translation, and author consultation. Cronbach's alpha was 0.91 in the original study^13^ and 0.78 in the current study.

### Data collection

The study was conducted in accordance with the principles of the Declaration of Helsinki. Data were collected via a Google Forms questionnaire, with participants recruited through the researchers’ social media platforms. The first page provided informed consent explaining the study's purpose and confidentiality. Those who agreed continued; others could not participate. While self-reporting may introduce bias and the cross-sectional design limits causal inference, anonymity and confidentiality were emphasized to mitigate these risks.

### Data analysis

Data were analyzed using IBM SPSS Statistics Version 26 (IBM Corp., Armonk, NY, USA). Descriptive statistics summarized sample characteristics. Data normality was assessed with the Kolmogorov-Smirnov test, as assumptions were met, independent t-tests and ANOVA were used for comparisons. Effect sizes were reported as Cohen's f and partial η^2^ for multiple-group comparisons, and Cohen's d for two-group comparisons, and were interpreted as small, medium, or large. Pearson correlations examined relationships between variables and scales. FGSIS was the dependent variable, FSFI-6 was the independent variable, with GRISS scores as a covariate controlling for sexual satisfaction. Age, education, and duration of marriage were added as control variables. FSFI-6's, GRISS's, and demographics’ contributions to FGSIS were assessed via multiple linear regression. Analyses (95%CI) reported β, SE, CI, R^2^, and significance, with p<0.05 considered significant.

### Ethical considerations

The study was approved by the Social and Human Sciences Ethics Committee of Yozgat Bozok University (Decision No: 12/61, dated 20.03.2024). Participants were informed that participation was voluntary and anonymous, no identifying information was collected, and only those who provided consent could proceed.

## RESULTS

In Table 1, 44.4% of women were university graduates, 72.1% had income equal to their expenses, 54.4% were housewives, 61.3% resided in the province, 50.4% had married after dating, 78.1% did not smoke, and 95.4% did not consume alcohol. In Table 1, significant differences in FGSIS scores were observed according to women's educational status and alcohol consumption (p=0.038, f=0.186, and 0.041, d=0.526, respectively), indicating small to medium effect sizes. FSFI-6 scores differed significantly based on women's education, income, employment status, place of residence, type of marriage, and alcohol consumption (respectively p=0.002, f=0.234; 0.001, f=0.198; <0.001, f=0.299; 0.002, f=0.188; <0.001, f=0.218; 0.012, d=0.646), with effect sizes ranging from small to medium-to-large. GRISS scores also showed significant differences according to women's place of residence and smoking habits (p=0.046, f=0.133; respectively 0.039, d=0.483), indicating small-to-medium effect sizes.

**Table 1:** Distribution of scale scores according to demographic and obstetric characteristics (n=351).

Characteristics		N	%	FGSIS	FSFI-6	GRISS
Education status						
	Literate	15	4.3	18.53±4.05^a^	18.73±5.56^ab^	53.60±16.74
	Primary school	32	9.1	19.16±3.11^a^	17.09±2.93^a^	51.09±10.50
	Secondary school	31	8.8	20.13±4.48^ab^	17.42±5.21^a^	58.97±14.03
	High school	87	24.8	20.29±4.08^ab^	19.63±5.05^ab^	56.95±16.94
	University	156	44.4	21.03±4.04^b^	20.37±4.84^b^	56.24±18.47
	Graduate	30	8.6	19.53±3.66^ab^	19.20±5.56^ab^	53.53±14.98
	Test stat.			2.383**	3.760**	1.751**
	P			**0.038**	**0.002**	0.133
	η^2^/f			**0.033/0.186**	**0.052/0.234**	0.014/0.119
Income status						
	Less than expenses	45	12.8	19.96±3.89	17.16±5.76^a^	56.71±15.38
	Equal to expenses	253	72.1	20.54±4.06	19.60±4.73^b^	55.71±16.99
	More than expenses	53	15.1	19.85±4.00	20.74±4.91^b^	55.75±17.27
	Test stat.			0.910**	6.875**	0.069**
	P			0.404	**0.001**	0.934
	η^2^/f			0.005/0.072	**0.038/0.198**	0.000/0.020
Employment status						
	Housewife	191	54.4	20.06±4.16	18.28±5.21^a^	55.52±16.10
	Officer	102	29.1	20.86±4.08	21.34±4.13^b^	54.64±17.40
	Private sector	53	15.1	20.38±3.43	20.34±4.42^b^	58.87±18.13
	Others	5	1.4	21.60±3.13	16.40±4.39^ab^	60.60±16.13
	Test stat.			1.047**	10.370**	0.905**
	P			0.372	**<0.001**	0.439
	η^2^/f			0.009/0.095	**0.082/0.299**	0.008/0.088
Place of residence						
	Province	215	61.3	20.68±4.10	20.07±4.91^b^	56.70±17.56^b^
	District	115	32.8	19.83±3.95	18.83±4.98^ab^	55.83±15.36^ab^
	Village	21	6.1	20.00±3.55	16.62±4.43^a^	47.19±14.24^a^
	Test stat.			1.797**	6.135**	3.103**
	P			0.167	**0.002**	**0.046**
	η^2^/f			0.010/0.102	**0.034/0.188**	**0.018/0.133**
Type of marriage						
	Arranged marriage	95	27.1	20.15±3.83	17.80±4.66^b^	56.44±15.87
	Dating	177	50.4	20.71±4.16	20.33±5.16^a^	56.90±18.05
	Arranged marriage+dating	79	22.5	19.84±3.92	19.49±4.46^ab^	52.75±14.63
	Test stat.			1.482**	8.297**	2.090**
	P			0.229	**<0.001**	0.126
	η^2^/f			0.008/0.092	**0.045/0.218**	0.010/0.100
Smoking status						
	Yes	77	21.9	20.99±4.24	19.87±5.68	59.81±19.55
	No	274	78.1	20.19±3.96	19.34±4.77	54.73±15.80
	Test stat.			1.545*	0.826*	2.094*
	P			0.123	0.409	**0.039**
	D			0.526	0.646	**0.483**
Alcohol use						
	Yes	16	4.6	22.38±4.62	22.50±4.05	63.56±17.70
	No	335	95.4	20.27±3.98	19.31±4.98	55.47±16.69
	Test stat.			2.056*	2.522*	1.889*
	P			**0.041**	**0.012**	0.060
	D			**0.526**	**0.646**	0.483

n: participants; %: percent; d: Cohen's d; f: Cohen's f; η^2^: eta squared.

a,b No significant differences were observed between groups assigned the same letter for each measurement., mean±SD;

* Independent-samples t test;

** One-Way ANOVA; FGSIS: Female Genital Self-Image Scale; FSFI-6: Female Sexual Function Index-6; GRISS: Golombok-Rust Inventory of Sexual Satisfaction.

Bold values indicate statistically significant results (p<0.05).

Table 2 shows significant positive correlations between GRISS scores, subscale means, and FGSIS (p<0.05). A negative relationship was observed between GRISS total scores and certain subscales (communication, satisfaction, and anorgasmia) with body height, as well as between the frequency subscale and FSFI scores (p<0.05). Positive correlations were observed between the frequency and avoidance subscales and the duration of marriage, and between the avoidance subscale and age (p<0.05). FGSIS was positively correlated with FSFI-6 and negatively correlated with age, body weight, and age at first pregnancy (p<0.05). FSFI-6 scores were negatively correlated with age and duration of marriage (p<0.05). Positive correlations were found between age and body weight, age and duration of marriage, body weight and duration of marriage, and duration of marriage and age at first pregnancy (p<0.05).

**Table 2:** Correlation between genital self-image, sexual function, sexual satisfaction, and other variables.

Variables	Mean±SD	1	2	3	4	5	6	7	8	9	10	11	12	13	14	15
1. GRISS	55.84±16.79	–														
2. Frequency	4.25±1.81	0.442**	–													
3. Communication	3.63±2.67	0.509**	0.182**	–												
4. Satisfaction	7.34±4.18	0.772**	0.267**	0.413**	–											
5. Avoidance	8.03±3.40	0.139**	0.232**	-0.152**	0.004	–										
6. Touch	9.51±4.55	0.706**	0.163**	0.290**	0.460**	-0.222**	–									
7. Vaginismus	8.17±2.85	0.559**	0.267**	0.246**	0.339**	0.206**	0.280**	–								
8. Anorgasmia	6.98±4.02	0.761**	0.245**	0.274**	0.502**	-0.003	0.488**	0.255**	–							
9. FGSIS	20.36±4.03	0.145**	-0.108*	0.132*	0.178**	-0.263**	0.229**	0.068	0.111*	–						
10. FSFI-6	19.46±4.98	0.033	-0.194**	-0.003	0.034	-0.291**	0.193**	0.061	0.064	0.298**	–					
11. Age	31.76±7.75	-0.024	0.105	0.053	-0.042	0.142**	-0.087	-0.027	-0.079	-0.128*	-0.183**	–				
12. Height	161.32±16.22	-0.114*	0.001	-0.120*	-0.109*	0.050	-0.063	-0.043	-0.111*	-0.091	-0.024	0.008	–			
13. Body weight	67.48±12.25	-0.009	0.001	0.004	-0.045	0.072	-0.026	0.032	-0.012	-0.105*	-0.078	0.253**	0.026	–		
14. Marriage duration	8.60±8.06	-0.025	0.119*	0.062	-0.017	0.124*	-0.087	-0.036	-0.083	-0.074	-0.207**	0.867**	-0.003	0.298**	–	
15. Age at first pregnancy	19.66±9.48	-0.051	0.004	0.048	-0.062	0.081	-0.043	-0.042	-0.105	-0.127*	-0.084	0.233**	0.044	0.096	0.200**	–

Pearson correlation analysis was conducted, mean±SD; FGSIS: Female Genital Self-Image Scale; FSFI-6: Female Sexual Function Index-6; GRISS: Golombok-Rust Inventory of Sexual Satisfaction; SD: standard deviation.

* p<0.05;

** p<0.01.

As shown in Table 3, the multiple regression analysis indicated that FSFI-6 score (B=0.228, SE=0.042, p<0.001, 95%CI 0.146, 0.310), age (B=-0.145, SE=0.053, p=0.006, 95%CI −0.249, −0.041), duration of marriage (B=0.139, SE=0.054, p=0.010, 95%CI 0.033, 0.244), and total GRISS score (B=0.032, SE=0.012, p=0.008, 95%CI 0.008, 0.056) were significant predictors of FGSIS scores. In contrast, the educational level did not significantly predict FGSIS (B=0.338, SE=0.183, p=0.065). The model accounted for 13.2% of the variance.

**Table 3:** The effect of sexual function, sexual satisfaction, and selected demographic variables on women's genital self-image (n=351).

	β^a^	β^b^	SE	Test stat.	p	LLCI	ULCI	R^2^
FSFI-6	0.282	0.228	0.042	5.479	**<0.001**	0.146	0.310	0.132
Age	-0.278	-0.145	0.053	-2.742	**0.006**	-0.249	-0.041	
Education status	0.106	0.338	0.183	1.851	**0.065**	-0.021	0.697	
Marriage duration	0.277	0.139	0.054	2.577	**0.010**	0.033	0.244	
GRISS	0.133	0.032	0.012	2.651	**0.008**	0.008	0.056	

β^a^: standardized coefficient; β^b^: nonstandardized coefficient; SE: standard error; R^2^: explained variance; LLCI: lower level confidence interval; ULCI: upper level confidence interval; FSFI-6: Female Sexual Function Index-6; GRISS: Golombok-Rust Inventory of Sexual Satisfaction. Statistically significant values are denoted in bold.

## DISCUSSION

The findings of this study, aimed at determining the effect of GSI on sexual function and sexual satisfaction in married women, are discussed in light of the existing literature.

In this study, women's GSI was significantly negatively associated with age, weight, and age at first pregnancy, consistent with the prior literature^7,14^. These associations likely reflect postpartum changes, societal aging stigma, and cumulative life experiences, suggesting that older age, higher weight, and later age at first pregnancy correspond to lower GSI. GSI appears to be shaped by multifactorial interactions and individual differences^7,14^.

In this study, GSI was positively associated with sexual satisfaction, suggesting that lower GSI relates to reduced satisfaction. Baltaci et al.^15^, similarly reported this relationship among pregnant women. GSI influences sexual satisfaction modestly^16^, with psychosocial and relational factors potentially^17^. However, a large-scale study by Komarnicky et al.^16^, found that GSI did not significantly predict sexual satisfaction among men, with the association being weak and largely nonsignificant. GSI is thought to influence sexual satisfaction, which is shaped by gender, psychosocial, and relational factors.

This study found that sexual function declined as sexual activity frequency increased, suggesting an unexpected relationship. Yazar et al.^18^, reported similar results, whereas other studies linked frequency to improved function and orgasm perception, emphasizing the roles of relationship status, health, and psychological well-being. In contrast, Huang et al.^19^ reported that masturbation frequency affected sexual function differently in single and partnered individuals, being more pronounced among singles. Sexual function may decrease in frequency of activity, and it is critically influenced by psychosocial and relational factors.

This study demonstrated a positive association between marital duration and sexual satisfaction, as also noted by Kaba and Egelioğlu Cetişli^20^. However, data from 3,207 German Pairfam participants revealed that marriage alone is not determinative, with some married individuals reporting lower sexual satisfaction than unmarried peers, underscoring the role of broader psychosocial determinants^21^.

This study identified a positive association between age and the avoidance subscale of sexual satisfaction. Avoidance may increase with age among women post-hysterectomy, influenced by aging and longer relationship duration^22^. Nonetheless, a Norwegian population-based study indicated that avoidance is more closely linked to sexual difficulties and the distress they generate, particularly lack of interest and general sexual distress, than to age itself^3^.

The study found a significant positive relationship between GSI and sexual function, consistent with the prior systematic reviews^23^. However, some studies report no association; for instance, an Iranian study found no differences between women with and without genital cosmetic surgery^8^. These findings suggest that cultural and individual factors shape the complex relationship between GSI and sexual function^8,24^.

Collectively, these findings, along with our study showing an association between GSI and both sexual function and satisfaction, suggest that GSI may have a role in sexual function and could be relevant in understanding sexual dysfunction, consistent with related studies^2,18,21^.

The 13.2% explained variance reflects the combined contribution of FSFI-6, age, marriage duration, and total GRISS scores to FGSIS scores, indicating a modest but meaningful effect. While Fudge and Byers reported that sexual comfort, general body image, and positive genital perception collectively accounted for 35% of variance in genital body image, the present findings indicate that GSI is only one of the multiple determinants^25^. Consistent with prior research, a positive GSI remains influential^15,25^, although other factors, including relationship quality, hormonal status, and psychosocial variables, likely contribute to sexual function and satisfaction^8,17,24^.

## CONCLUSION

This study found that women's GSI is associated with sexual function and satisfaction, though the effect is modest. Marriage duration, age, weight, and age at first pregnancy also impact GSI. Clinically, healthcare providers should implement targeted strategies: body-positive counseling, partner-inclusive therapies, educational programs, and body-image workshops to optimize women's sexual well-being.

### Limitations

This cross-sectional study precludes causal inferences and relies on self-reported data, potentially subject to social desirability and recall biases, limiting generalizability. A longitudinal follow-up incorporating qualitative interviews is recommended.

## Data Availability

The datasets generated and/or analyzed during the current study are available from the corresponding author upon reasonable request.
